# Supplementary material for: Quality Characteristics of Karst Plateau Tea (Niaowang) in Southwest China and Their Relationship with Trace Elements
Source: Toxics. 2023 Jun 2;11(6):502. doi: 10.3390/toxics11060502 (PMC10305230; doi:10.3390/toxics11060502)
Supplement: Supplementary file 1 [file toxics-2423580-supplementary.pdf › toxics-2423580-supplementary.pdf]

As shown in table S1, The content of trace elements in Niaowang tea fluctuates significantly with the seasons, and the content in autumn is higher than that in summer. The highest content difference in summer and autumn is As, up to 18.18 times, and the lowest is Zn, up to 1.27 times. At the same time, the content of each element in Niaowang tea is significantly different, the lowest content is As, followed by Hg, Se, Pb, Ni, Zn, Fe, and the highest content is Mn. The average content of Mn is 250 mg/kg in summer and 423.43 mg/kg in autumn, with the highest content of 1490 mg/kg and the lowest content of 39.84 mg/kg. Drinking tea is the main way for people to absorb Mn. Niaowang tea is rich in Mn, which can well meet people's demand for Mn. Compared with the limited contents of Pb, As and Hg in tea in <<NY 659-2003>> and <<GB 2762-2017>>, the contents of As and Hg are lower than the limit value, indicating that there is no As and Hg pollution in Niaowang tea. However, Pb in autumn exceeds the national limit, indicating that there is certain Pb pollution in autumn. The content of trace elements in tea was determined by sampling the Niaowang tea in Yunwu Town, Guiding County in three seasons. It was found that the trace elements changed significantly with the season. This may be because the trace elements in the atmospheric sediment entered the tea after polluting the soil and water source during the growth of the tea tree, resulting in the seasonal change of the content of trace elements in the tea.

Table S1. The contents of 8 trace elements in Niaowang tea

	**As**		**Hg**		**Se**		**Pb**	
	Summer	Autumn	Summer	Autumn	Summer	Autumn	Summer	Autumn
Mean	0.02	0.28	0.12	0.25	0.14	0.97	1.25	13.43
SD	0.01	0.13	0.04	0.15	0.06	0.23	0.34	3.25
Limit number	2.00	2.00	0.30	0.30	——	——	5.00	5.00
Maximum	0.13	0.59	0.34	1.17	0.31	2.35	5.45	32.08
Minimum	0.00	0.00	0.00	0.00	0.04	0.22	0.00	2.99
Sampling Number	28.00	28.00	28.00	28.00	28.00	28.00	28.00	28.00

	**Ni**		**Zn**		**Fe**		**Mn**	
	Summer	Autumn	Summer	Autumn	Summer	Autumn	Summer	Autumn
Mean	3.55	8.86	23.66	30.07	50.58	251.25	250.30	423.43
SD	1.23	3.22	6.73	8.36	10.35	69.35	55.47	120.35
Limit number	——	——	——	——	——	——	——	——
Maximum	7.33	23.90	40.19	98.90	192.30	1112.00	1286.00	1490.00
Minimum	0.00	2.92	4.49	9.85	11.90	70.90	39.84	46.70
Sampling Number	28.00	28.00	28.00	28.00	28.00	28.00	28.00	28.00
